# The prevalence of intestinal nematodes among red foxes (*Vulpes vulpes*) in north-western Poland

**DOI:** 10.1186/s13028-021-00584-0

**Published:** 2021-05-05

**Authors:** Agnieszka Tylkowska, Bogumiła Pilarczyk, Agnieszka Tomza-Marciniak, Renata Pilarczyk

**Affiliations:** 1 Department of Animal Environment Biology, Faculty of Animal Breeding, Bioengineering and Conservation, Warsaw University of Life Sciences, Ciszewskiego 8, 02-787 Warsaw, Poland; 2 Department of Animal Reproduction Biotechnology and Environmental Hygiene, Faculty of Biotechnology and Animal Husbandry, West Pomeranian University of Technology, Szczecin, Poland; 3 Laboratory of Biostatistics, Faculty of Biotechnology and Animal Husbandry, West Pomeranian University of Technology, Szczecin, Poland

**Keywords:** Ecological indicators, Helminths, Nematodes, Prevalence, Red fox

## Abstract

**Background:**

The red fox (*Vulpes vulpes*) is widely distributed in the Northern Hemisphere and Australia. The presence of nematode-infected foxes in urbanized areas increases the risk of transmission of nematodes to domestic dogs and thus, to humans. The aim of this study was to determine the prevalence and species composition of intestinal nematodiasis in red foxes in Western Pomerania, a province in north-western Poland. The intestinal contents of 620 red foxes killed during a government reduction shooting programme were examined for adult nematodes using the sedimentation and counting technique (SCT).

**Results:**

Intestinal nematodes, including *Toxocara canis*, *Toxascaris leonina*, *Uncinaria stenocephala* and *Trichuris vulpis*, were found in 77.3% (95% CI 73.8–80.4%) of the examined foxes with a mean infection burden of 20.1 nematode per animal. Male and female foxes had similar infection burdens.

**Conclusions:**

The nematodes are present in high prevalence and intensity among foxes in north-western Poland. Furthermore, this high prevalence of nematodes in foxes may likely constitute a health risk to humans and domestic animals due to increasing fox densities in urban and periurban areas.

## Background

The red fox (*Vulpes vulpes*) is widely distributed in the Northern Hemisphere and Australia [1]. A rapid increase in its number has been observed in many European countries since the late twentieth century [2–4]. This growth may be explained by a reduction in mortality rate due to intensive rabies vaccination campaigns and the opportunistic behaviour of the fox itself [2].

The growth in the fox population has resulted in its expansion to new habitats. Unfortunately, as foxes may be infected by zoonotic intestinal nematodes, their presence in urbanized areas increases the risk of transmission of these nematodes to humans, either directly, or indirectly through infection of domestic dogs [5–7]. Infected foxes can contaminate sandpits, parks and squares with their faeces, thus exposing humans to the eggs of zoonotic nematode species such as *Toxocara canis*, *Toxascaris leonina* and *Trichuris vulpis*, and nematodes from the Ancylostomatidae family [8–12].

The most widespread helminth zoonosis in developed countries is toxocariasis, known to cause visceral and ocular larva migrans syndrome [3, 13, 14]. In humans, as paratenic hosts, the larvae of *T. canis*, responsible for toxocariasis, do not develop into an adult stage but migrate throughout the tissues and remain as L3 arrested larvae for an extended period of time [15].

The prevalence of *T. canis* seropositive cases in humans has been estimated at 2.4% in Denmark [16], 7% in Sweden [17] and 6.3% in Austria [18]. A significant proportion of the human population in Poland have also been found to harbour *T. canis* antibodies, with a seroprevalence ranging from 18.6 to 43% depending on the region [19, 20]. Similar symptoms can be observed following infection with *T. leonina* and *T. vulpis* larvae [21].

An additional threat is also posed to humans by hookworms, i.e. nematodes of the Ancylostomatidae family, such as *Ancylostoma caninum* and *Uncinaria stenocephala*, with approximately 1.3 billion people being estimated to be infected by a nematode from this family [22]. Hookworm infection rarely results in mortality but leads to anaemia and malnutrition [23], which lead to chronic health problems such as lethargy and impaired physical and cognitive development [24].

A number of studies, including one carried out from 1996 to 1998 in north-western Poland by Bieńko [25], indicate that the occurrence of nematodes in foxes varies considerably [26–30]. Therefore, the aim of the present study was to determine the prevalence of intestinal nematoidiasis in red foxes (*Vulpes vulpes*) in north-western Poland and investigate other parameters associated with its presence, e.g. examine its distribution between sexes and predilection.

## Methods

The study was conducted in Western Pomerania, a province of north-western Poland. The material comprised a total of 620 red foxes (236 females and 384 males) culled during a government reduction shooting programme in 18 counties in the region. The remains of the foxes were acquired from the Departments of Veterinary Hygiene in Szczecin and Koszalin during the hunting seasons of 2008/2009, 2009/2010 and 2010/2011. The minimum sample size was calculated according to the Cochran formula.

Parasitological analyses were carried out at the Department of Veterinary Hygiene in Szczecin and Koszalin. Fox carcasses delivered to the department by hunters were initially frozen at − 70 °C for four days to prevent transmission of infective *Echinococcus multilocularis* eggs. The carcasses were then thawed and examined.

The alimentary tracts of the foxes were subjected to parasitological analysis using sectional methods described by Eckert et al. [31, 32] and by Stefański and Żarnowski [33]. Briefly, the alimentary tract samples were isolated from the animals and divided into oral cavity, oesophagus, stomach, small intestine, caecum and large intestine samples. In addition, the small intestine was further divided into three sections: duodenum, jejunum and ileum. The organs were opened along their entire length. Visible parasites were removed immediately from the mucosa and placed in Petri dishes with water. Other parasites were collected using the sedimentation and counting technique (SCT). The sex of the animals was identified based on the gonads. The recovered nematodes were identified according to Stefański and Żarnowski [33] and preserved in 70% alcohol.

The Jaccard coexistence index (J) [34] was used to compare the coexistence of particular species of nematodes, where the feature is the presence of a species in the sample (environment). A value ​​of 100% indicates that two analysed nematode species always co-occur, regardless of their numbers, and 0% indicates that the two species are never observed together.

$$ Jp1p2 = \;\frac{c}{a + b - c}\; \times \;100$$

where: Jp1p2—coexistence index between species p1 and p2; a—the number of occurrences of species p1; b—the number of occurrences of species p2; c—the number of common occurrences of species p1 and p2 in the habitat.

The obtained results were analysed using Statistica 10.0. The occurrence of individual nematode species was compared between the sex of the host was determined using the Mann–Whitney U-test. The occurrence of nematodes was compared between individual sections of the gastrointestinal tract using the Kruskal–Wallis test. The confidence interval of a proportion was calculated by the modified Wald method, as recommended by Agresti and Coull [35].

## Results

Nematodes were found in 77.3% (95% CI 73.8–80.4%) of analysed foxes, while the mean infection intensity was 20.1 nematodes per animal. *T. canis*, *T. leonina*, *U. stenocephala* and *T. vulpis* were isolated from the tested foxes. Of these, *U. stenocephala* was the most prevalent (34.0%, 95% CI 30.4–37.9%), with an intermediate intensity of infection (20.7) (Table 1).

**Table 1:** Parameters of occurrence of nematodes in red fox

Parasite	Number of foxes infected / tested	Prevalence (%) (95% CI)	Intensity of infection	
			Mean	Range
*Toxocara canis*	187/620	30.2 (26.7–33.9)	12.2	1–84
*Toxascaris leonina*	163/620	26.3 (23.0–30.0)	16.9	1–97
*Uncinaria stenocephala*	211/620	34.0 (30.4–37.9)	20.7	1–96
*Trichuris vulpis*	74/620	11.9 (9.6–14.7)	3.0	1–15
Nematodes (total)	479/620	77.3 (73.8–80.4)	20.1	1–140

*T. canis* was more commonly observed in male foxes (32.0%, 95% CI 27.6–36.9%) than females (27.1%, 95% CI 21.8–33.1%), while *U. stenocephala* was more common in female foxes (37.7%, 95% CI 31.8–44.0%) than males (31.8%, 95% CI 27.3–36.6%). These differences in incidence were not statistically significant (p > 0.05). In both males and females, the highest mean intensity of infection was found for *U. stenocephala*, which displayed similar intensities in female (21.7) and male foxes (19.9) (Table 2).

**Table 2:** Incidence of nematodes in red foxes with regard to sex of host

Parasite	Sex of host	Number of foxes infected / tested	Prevalence (%) (95% CI)	Mann–Whitney *U*-test value	Intensity of infection		
					Mean	Range	Mann–Whitney *U*-test value
*Toxocara canis*	♂	123/384	32.0 (27.6–36.9)	Z = − 0.44 p = 0.660	12.0	1–84	Z = − 1.37 p = 0.172
	♀	64/236	27.1 (21.8–33.1)		12.6	1–73	
*Toxascaris leonina*	♂	106/384	27.6 (23.4–32.3)	Z = − 0.44 p = 0.662	15.8	1–65	Z = − 0.80 p = 423
	♀	57/236	24.2 (19.1–30.0)		18.8	1–97	
*Uncinaria stenocephala*	♂	122/384	31.8 (27.3–36.6)	Z = − 0.58 p = 0.560	19.9	1–96	Z = − 1.69 p = 0.091
	♀	89/236	37.7 (31.8–44.0)		21.7	1–91	
*Trichuris vulpis*	♂	42/384	10.9 (8.2–14.5)	Z = − 0.22 p = 0.828	2.7	1–15	Z = − 1.08 p = 0.280
	♀	32/236	13.6 (9.7–18.6)		3.4	1–15	
Nematodes (total)	♂	302/384	78.7 (74.3–82.5)	Z = − 0.97 p = 0.333	18.9	1–100	Z = − 0.15 p = 0.878
	♀	177/236	75.0 (69.1–80.1)		22.2	1–140	

Nematodes were observed in the duodenum, jejunum, ileum and caecum; however, none were noted in the oral cavity, the oesophagus, the stomach or the large intestine. The parts of the alimentary tract where nematodes were found were then subjected to further analysis; among these, significantly higher numbers of nematodes were found in the jejunum than in the duodenum or ileum (Kruskal–Wallis; H = 96.7; p = 0.000) (Table 3).

**Table 3:** The occurrence of nematodes in red foxes according to location in the intestine

Parasite	Part of the small intestine	Number of foxes infected / tested	Prevalence (%) (95% CI)	Kruskal–Wallis value	Intensity of infection		
					$\bar x$	Range	Kruskal–Wallis value
*Toxocara canis*	Duodenum	128/620	20.7 (17.6–24.0)	H = 50.6** p < 0.001	3.2	1–23	H = 61.7** p < 0.001
	Jejunum	161/620	26.0 (22.7–29.6)		9.8	1–50	
	Ileum	64/620	10.3 (8.2–13.0)		4.5	1–25	
*Toxascaris leonina*	Duodenum	122/620	19.7 (16.7–23.0)	H = 52.0** p < 0.001	3.5	1–20	H = 88.4** p < 0.001
	Jejunum	147/620	23.7 (20.5–27.2)		14.4	1–79	
	Ileum	54/620	8.7 (6.7–11.2)		3.9	1–12	
*Uncinaria stenocephala*	Duodenum	128/620	20.7 (17.6–24.0)	H = 55.8** p < 0.001	4.3	1–28	H = 109.3** p < 0.001
	Jejunum	196/620	31.6 (28.1–35.4)		16.3	1–72	
	Ileum	88/620	14.2 (11.7–17.2)		7.0	1–35	
*Trichuris vulpis*	Caecum	74/620	11.9 (9.6–14.7)	H = – p = –	3.0	1–15	H = – p = –
Nematodes (total)	Duodenum	378/620	61.0 (57.1–64.7)	H = 96.7** p < 0.001	3.7	1–28	H = 357.5** p < 0.001
	Jejunum	504/620	81.3 (78.0–84.2)		13.7	1–108	
	Ileum	280/620	45.2 (41.3–49.1)		4.8	1–48	

Presence of statistical significance: ^*^Statistically significant at p ≤ 0.05, ^**^Statistically significant at p ≤ 0.01

Regarding *T. canis*, its numbers differed significantly between the duodenum and the ileum and between the jejunum and the ileum (Kruskal–Wallis; H = 50.6; p < 0.001). In addition, the occurrence of *T. leonina* significantly differed between the duodenum and the ileum, and between the jejunum and the ileum (Kruskal–Wallis; H = 52.0; p < 0.001), and *U. stenocephala* differed significantly between the duodenum and the jejunum, and between the jejunum and the ileum (Kruskal–Wallis; H = 55.8; p < 0.001) (Table 3).

In the duodenum, the most common nematodes were *U. stenocephala* (20.7%, 95% CI 17.6–24.0%) and *T. canis* (20.7%, 95% CI 17.6–24.0%); however, the highest mean intensity of infection was found for *U. stenocephala* (4.3) (Table 3). Of the identified nematodes, the prevalence and the highest mean intensity of infection were noted for *U. stenocephala* in the jejunum (31.6% [95% CI 28.1–35.4%], 16.3, respectively) and the ileum [14.2% (95% CI 11.7–17.2%), 7.0, respectively] (Table 3). *T. vulpis* (11.9%, 95% CI 9.6–14.7%) was found only in the caecum (Table 3).

Our findings indicate variations in both the chance of encountering certain pairs of nematode species and the strength of the relationship between them, as measured by the Jaccard index (J). The most common pairs comprised *U. stenocephala* and *T. canis* (66 joint occurrences, J = 14.2%) and *U. stenocephala* and *T. leonina* (40 joint occurrences, J = 9.7%) (Table 4).

**Table 4:** Frequency of coexistence of individual nematode species in a single host organism

Parasite	*Toxocara canis*	*Toxascaris leonina*	*Uncinaria stenocephala*	*Trichuris vulpis*
*Toxocara canis*	187^a^	3.8^c^	14.2^c^	6.5^c^
*Toxascaris leonina*	14^b^ (2.3)	163^a^	9.7^c^	6.7^c^
*Uncinaria stenocephala*	66^b^ (10.7)	40^b^ (6.5)	211^a^	7.2^c^
*Trichuris vulpis*	18^b^ (2.9)	17^b^ (2.7)	22^b^ (3.6)	74^a^

^a^The number of foxes in which the species of nematode was found;

^b^The number of co-occurring nematode species (prevalence);

^c^The Jaccard coexistence index between the species of nematode (%)

## Discussion

The most frequently-observed gastro-intestinal nematode in the red foxes examined in the present study was *U. stenocephala*. This finding is in line with previous studies on red foxes in Europe in general [3, 36–39] and in studies in northern and southern Poland [40, 41]. However, other studies conducted in western and southern Poland showed a significantly lower prevalence, ranging from 11 to 35% [42–44]. A higher prevalence of *U. stenocephala* has been found in Estonia (84.3%) [45], Lithuania (76.9%) [46] and Italy (75.4%) [37]; however, *U. stenocephala* are less common in Ukraine (27.1%) [47], Romania (15.0%) [48], the Czech Republic (10.0%) [2], Switzerland (5.3%) [6] and Norway (1.6%) [49].

The prevalence of *U. stenocephala* in foxes has been found to increase in recent years, from 39.0% in 2008 to 65.0% in 2010 in Sweden [50], and from 68.6% in 2006 to 84.1% in 2013 in Denmark [36, 51].

In the present study, *T. canis* was more common than *T. leonina*. A similar trend was observed in earlier studies in the same region, with the frequency of *T. leonina* infection increasing more than 12-fold over the test period [25]; however, the prevalence of *T. canis* was found to be higher than in the present study (42.3%). This situation could be caused by the availability of food, mostly rodents, in the environment occupied by the foxes. Okulewicz et al. [52] highlight the role of small rodents as paratenic hosts of nematodes and note that rodents may contribute significantly to the spread of *T. canis* and *T. leonina* in the environment. Pucek et al. [53] report that most species of rodents display cycles characterised by periodic and rapid changes in population density. In the case of forest mice and voles, their numbers are directly influenced by mast years of deciduous tree species (oak, beech, hornbeam) and weather conditions in the winter season [54].

The abundance of *T. canis* and *T. leonina* in foxes is also influenced by the growing number of animals infected with the parasites, the large numbers of eggs excreted into the environment, and the considerable resistance of the eggs to adverse environmental conditions. Given appropriate temperature and humidity conditions, eggs are known to remain infective for at least a year [54]. Otranto and Deplazes [55] note that *T. canis* infections are generally higher in young foxes, i.e. under six months of age, although adult foxes with weak immunity also demonstrate a relatively high infection rate by intestinal parasites in endemic areas.

A higher prevalence of *T. canis* than *T. leonina* has also been found in other countries, including Denmark (59.4 and 0.6%) [51], Italy (52.6 and 0%) [37], Slovakia (25.8 and 17.5%) [56], and Kyrgyzstan (30.4 and 5.9%, respectively) [57]. However, *T. leonina* has been found to predominate over *T. canis* in other countries, such as Sweden (65.0 and 30.0%) [50], Turkey (65.0 and 20.0%) [58], and Norway (12.9 and 1.6%, respectively) [49]. An exception was the Czech Republic, where both species were present in foxes in equal numbers [2].

The most rarely-found parasite in this study was *T. vulpis*. This finding confirms the results of Karamon et al. [40] from Poland, who estimate its prevalence among foxes to be 1.3% in south-eastern Poland and 4.4% in northern Poland. A high prevalence (64.4%) has only been reported in southern Poland [59]. Interestingly, this parasite has not been found in central Poland [60].

In other European countries, the occurrence of *T. vulpis* among foxes varies considerably. In Italy, 12.3% of tested foxes were found to be infected [37], while the prevalence was found to 7.7% in Sweden, 5.5% in Switzerland, 4.8% in Ukraine, and 1.6% in Norway [6, 37, 47, 49]. However, as surveillance in foxes is often limited to the small intestine, *T. vulpis*, which is localized in the large intestine, is not typically considered during post-mortem parasitological investigations [40].

In the present study, similar nematode prevalence levels were observed between male and female foxes. Similarly, previous studies have also reported no sex-related differences in prevalence [2, 41]. Vervaeke et al. [61] suggest that these observed lack of sex-driven differences in the prevalence of nematodes may result from the fact that both male and female foxes feed on the same type of food. Their main diet is rodents, which are intermediate hosts of many parasite species. However, the higher prevalence of *T. canis* observed in males in the present study might be a result of behavioural differences between the sexes. Male foxes are more active than females, and their hunting grounds are larger than those of females. Moreover, males are larger than females, and have higher food requirements [62].

Unfortunately, it is difficult to find suitable comparisons for our results regarding the distribution of parasites in the digestive tract of the studied foxes, as literature in this area is scarce. Based on the present findings, the nematodes *T. canis* and *T. leonina* prefer to colonize the duodenum and jejunum and are significantly less commonly observed in the ileum. *U. stenocephala* colonizes the jejunum far more readily than the duodenum or ileum. One exception is *T. vulpis*, which was observed only in the caecum, suggesting that this may be the predilection site of the parasite. A similar prevalence was found by Fiocchi et al. [37] in the caeca of seven foxes (12.3%).

*U. stenocephala* was found to co-occur most often with *T. canis* (J = 14.2%) and less often with *T. leonina* (J = 9.7%). *T. canis* and *T. leonina* were found to co-occur in only 14 foxes (J = 3.8%). Therefore, the high prevalence of *T. canis* in foxes and pets may lead to a low prevalence of *T. leonina* and vice versa [50]; however, Jankovska et al. [2] report the co-occurrence of these two nematode species in 70.6% of tested foxes in the Czech Republic.

The differences observed in the prevalence of nematodes between specific European countries might be an effect of the variable diet of the foxes, which depends on the geographical location and species variability of prey in a given region [62]. Foxes prefer to hunt for small rodents in their hunting range. This is particularly noted in areas with large populations of some rodent species, such as voles; for example, in western and northern Poland, markedly greater consumption of voles and other field rodents may be observed in the summer and autumn seasons. In central and eastern Poland, however, the winter diet of foxes is dominated by rodents, hares and carrion; hares and birds dominate in spring, and large amounts of fruit appear in the diet in summer and autumn [63]. In places where voles are absent, e.g., in Ireland, foxes readily eat other rodent species, such as rats [64]. Carrion is a regular component of the diet of foxes, irrespective of their place of occurrence; in fact, under specific circumstances, carrion may constitute almost half of their diet [62]. A substantial share of carrion in the diet is observed in mountain areas and forests in the Northern Hemisphere because thick and long-lasting snow cover makes catching small rodents difficult [65].

The foxes inhabiting north-western Poland demonstrate are frequently hosts to nematodes. A number of studies suggest that the prevalence of geohelminths is associated with climate conditions, *inter alia* temperature, air and soil humidity, and mean annual rainfall, which extend the lifespan of their free-living developmental stages [2, 40, 66–68]. The region of north-western Poland is particularly suitable for the development of parasites, being situated close to the sea, with substantial forest cover and a temperate climate, a total annual rainfall of 500–800 mm, a mean annual temperature of 7–9 °C and high humidity. Such conditions certainly increase the threat to the health of humans and pets by increasing the risk of parasite transmission. This risk is further increased by the growing number of foxes searching for new places to live in areas inhabited by humans.

## Conclusions

Our findings demonstrate that nematodes are present in high prevalence and intensity among foxes in north-western Poland. Furthermore, this high prevalence of nematodes in foxes may likely constitute a health risk to humans and domestic animals due to increasing fox densities in urban and periurban areas.

## Data Availability

The datasets used and/or analysed during the current study are available from the corresponding author on request.

## Abbreviations

J: The Jaccard coexistence index

## References

[1] Hoffmann M, Sillero-Zubiri C. *Vulpes vulpes*. The IUCN Red List of Threatened Species. 2016.

[2] Jankovská I, Brožová A, Matějů Z, Langrová I, Lukešová D, Sloup V (2016). Parasites with possible zoonotic potential in the small intestines of red foxes (*Vulpes vulpes*) from Northwest Bohemia (CzR). Helminthol.

[3] Magi M, Guardone L, Mignone W, Prati MC, Macchioni F (2016). Intestinal helminths of red foxes (*Vulpes vulpes*) in north-west Italy. Helminthol.

[4] Veronesi F, Morganti G, Di Cesare A, Lepri E, Cassini R, Zanet S (2014). *Eucoleus boehmi* infection in red fox (*Vulpes vulpes*) from Italy. Vet Parasitol.

[5] Gloor S, Bontadina F, Hegglin D, Deplazes P, Breitenmoser U (2001). The rise of urban fox population in Switzerland. Mamm Biol.

[6] Koller B, Hegglin D, Schnyder M (2019). A grid-cell based fecal sampling scheme reveals: land-use and altitude affect prevalence rates of *Angiostrongulus vasorum* and other parasites of red foxes (*Vulpes vulpes*). Parasitol Res.

[7] Reperant LA, Hegglin D, Fischer C, Kohler L, Weber JM, Deplazes P (2007). Influence of urbanization on the epidemiology of intestinal helminths of the red fox (*Vulpes vulpes*) in Geneva, Switzerland. Parasitol Res.

[8] Lotsch F, Vingerling R, Spijker R, Grobusch MP (2017). Toxocariasis in humans in Africa—a systematic review. Travel Med Infect Dis.

[9] Maleki B, Khorshidi A, Gorgipour M, Mirzapour A, Majidiani H, Foroutan M (2018). Prevalence of *Toxocara* spp. Eggs in soil of public areas in Iran: a systematic review and meta-analysis. Alexandria J Med..

[10] Nijsse R, Mughini-Gras L, Wagenaar JA, Franssen F, Ploeger HW (2015). Environmental contamination with *Toxocara* eggs: a quantative approach to estimate the relative contributions of dogs, cats and foxes, and to assess the efficacy of advised interventions in dogs. Parasit Vectors.

[11] Ondriska F, Macuhova K, Melicherova J, Reiterova K, Valentova D, Beladicova V (2013). Toxocariasis in urban environment of western Slovakia. Helminthol.

[12] Papajova I, Pipkova J, Papaj J, Cizmar A (2014). Parasitic contamination of urban and rural environments in the Slovak Republic: dog’s excrements as a source. Helminthol.

[13] Aghamolaie S, Seyyedtabaei SJ, Behniafar H, Foroutan M, Saber V, Hanifehpur H (2019). Seroepidemiology, modifiable risk factors and clinical symptoms of *Toxocara* spp. infection in northern Iran. Trans R Soc Trop Med Hyg..

[14] Zibaei M (2017). Helminth infections and cardiovascular diseases: *Toxocara* species in contributing to the disease. Curr Cardiol Rev.

[15] Strube C, Heuer L, Janecek E (2013). *Toxocara* spp. infection in paratenic hosts. Vet Parasitol..

[16] Stensvold CR, Skov J, Moller LN, Jensen PM, Kapel CMO, Petersen E (2009). Seroprevalence of human toxocariasis in Denmark. Clin Lab Immunol.

[17] Ma G, Holland CV, Wang T, Hofmann A, Fan CK, Maizels RM (2018). Human toxocariasis. Lancet Infect Dis.

[18] Poeppl W, Herkner H, Tobudic S (2013). Exposure to *Echinococcus multilocularis*, *Toxocara canis*, and *Toxocara cati* in Austria: a nationwide cross-sectional seroprevalence study. Vector Borne Zoonotic Dis.

[19] Hermanowska-Szpakowicz T, Kondrusik M, Świerzbińska R, Zajkowska J, Pancewicz S (2001). Incidence of antibody detection against *Toxocara canis* and clinical symptoms in inhabitants of North-Eastern Poland. Pol Merkur Lekarski.

[20] Balicka-Ramisz A, Horodnicka-Jóźwa A, Ramisz A, Laurans Ł, Wnuk W, Pilarczyk B (2005). Toxocariasis in dogs and the presence of antibodies against *Toxocara canis* in children. Med Wet.

[21] Despommier D (2003). Toxocariasis: clinical aspects, epidemiology, medical ecology and molecular aspects. Clin Microbiol Rev.

[22] Chan MS (1997). The global burden of intestinal nematode infections—fifty years on. Parasitol Today.

[23] Hotez PJ, Bethony JM, Diemert DJ, Pearson M, Loukas A (2010). Developing vaccines to combat hookworm infection and intestinal schistosomiasis. Nat Rev Microbiol.

[24] Hotez PJ, Brooker S, Bethony JM, Bottazzi ME, Loukas A, Xiao S (2004). Hookworm infection. N Engl J Med.

[25] Bieńko R. Research on parasitofauna and the role of a free living foxes as a reservoir of parasite zoonoses in north-western Poland. Szczecin: Agricultural University (dissertation); 2000.

[26] Andras T (2001). Data on the parasitological status of the red fox in Hungary. Mag Allat Lapja.

[27] Cisek A, Ramisz A, Balicka-Ramisz A, Pilarczyk B, Laurans Ł (2004). The extensity of *Toxocara canis* (Werner, 1782) in dogs and foxes in north-western Poland. Wiad Parazytol.

[28] Criado-Fornelio A, Gutierrez-Garcia L, Rodriguez-Caabeiro F, Reus-Garcia E, Roldan-Soriano MA, Diaz-Sanchez MA (2000). A parasitological survey of wild red foxes (*Vulpes vulpes*) from the province of Guadalajara. Spain Vet Parasitol.

[29] Di Cerbo AR, Manfredi MT, Bregoli M, Ferro N, Cova M (2008). Wild carnivores as source of zoonotic helminths in north-eastern Italy. Helminthol.

[30] Zoltan S, Tamas S, Istvan V (2004). Prevalence, veterinary and public health aspects of gastrointestinal parasites in red foxes (*Vulpes vulpes*) in Hungary. Magy Allatorvosok.

[31] Eckert J, Deplazes P, Ewald D, Gottstein B (1991). Parasitological and immunological methods of detecting *Echinococcus multilocularis* in foxes. Mitt Österr Ges Tropenmed Parasitol.

[32] Eckert J, Kutzer E, Rommel M, Bürger HJ, Körting W (1992). Veterinary parasitology.

[33] Stefański W, Żarnowski E (1971). Recognition of parasitic invasions in animals.

[34] Magurran AE (2004). Measuring biological diversity.

[35] Agresti A, Coull BA (1998). Approximate is better than "exact" for interval estimation of binomial proportions. Am Stat.

[36] Al-Sabi MN, Chriél M, Jensen TH, Enemark HL (2013). Endoparasites of the raccoon dog (*Nyctereutes procyonoides*) and the red fox (*Vulpes vulpes*) in Denmark 2009–2012—a comparative study. Int J Parasitol Parasites Wildl.

[37] Fiocchi A, Gustinelli A, Gelmini L, Rugna G, Renzi M, Fontana MC (2016). Helminth parasites of the red fox *Vulpes vulpes* (L., 1758) and the wolf *Canis lupus italicus* Altobello, 1921 in Emilia-Romagna, Italy. Ital J Zool..

[38] Rataj AV, Posedi J, Zele D, Vengust G (2013). Intestinal parasites of the red fox (*Vulpes vulpes*) in Slovenia. Acta Vet Hung.

[39] Schuster RK, Shimalov VV (2017). A comparative study of helminths of raccoon dogs (*Nyctereutes procynoides*) and red foxes (*Vulpes vulpes*) sharing the same territory. Asian Pac J Trop Dis.

[40] Karamon J, Dąbrowska J, Kochanowski M, Samorek-Pieróg M, Sroka J, Różycki M (2018). Prevalence of intestinal helminths of red foxes (*Vulpes vulpes*) in central Europe (Poland): a significant zoonotic threat. Parasit Vectors.

[41] Karamon J, Samorek-Pieróg M, Moskwa B, Różycki M, Bilska-Zając E, Zdybel J (2016). Intestinal helminths of racoon dogs (*Nyctereutes procyonoides*) and red foxes (*Vulpes vulpes*) from the Augustów Primeval Forest (north-eastern Poland). J Vet Res.

[42] Balicka-Ramisz A, Ramisz A, Pilarczyk B, Bieńko R (2003). Fauna of gastro-intestinal parasites in red foxes in western Poland. Med Wet.

[43] Borecka A, Gawor J, Malczewska M, Malczewski A (2009). Prevalence of zoonotic helminth parasites of the small intestine in red foxes from central Poland. Med Wet.

[44] Pacoń J, Sołtysiak Z, Nicpoń J, Janczak M (2006). Prevalence of internal helminths in red foxes (*Vulpes vulpes*) in selected regions of Lower Silesia. Med Wet.

[45] Laurimaa L, Moks E, Soe E, Valdmann H, Saarma U (2016). *Echinococcus multilocularis* and other zoonotic parasites in red foxes in Estonia. Parasitol.

[46] Bruzinskaite-Schmidhalter R, Sarkunas M, Malakauskas A, Mathis A, Torgerson PR, Deplazes P (2012). Helminths of red foxes (*Vulpes vulpes*) and raccoon dogs (*Nyctereutes procyonides*) in Lithuania. Parasitol.

[47] Varodi EI, Malega AM, Kuzmin YI, Kornyushin VV (2017). Helminths of wild predatory mammals of Ukraine. Nematodes Vestnik Zoologii.

[48] Siko SB, Deplazes P, Ceika C, Tivadar CS, Gogolin I, Popescu S (2011). *Echinococcus multilocularis* in south-eastern Europe (Romania). Parasitol Res.

[49] Myskova E, Broz M, Fuglei E, Kvicerova J, Macova A, Sak B (2019). Gastrointestinal parasites of arctic foxes (*Vulpes lagopus*) and sibling voles (*Microtus levis*) in Spitsbergen. Svalbard Parasitol Res.

[50] Meijer T, Mattsson R, Angerbjörn A, Osterman-Lind E, Fernandez-Aguilar X, Gavier-Widen D (2011). Endoparasites in the endangered Fennoscandian population of arctic foxes (*Vulpes lagopus*). Eur J Wildlife Res.

[51] Saeed I, Maddox-Hyttel C, Monrad J, Kapel CMO (2006). Helminths of red foxes (*Vulpes vulpes*) in Denmark. Vet Parasitol.

[52] Okulewicz A, Perec-Matysiak A, Buńkowska K, Hildebrand J (2012). *Toxocara canis, Toxocara cati* and *Toxascaris leonina* in wild and domestic carnivores. Helminthol.

[53] Pucek Z, Jędrzejewski W, Jędrzejewska B, Pucek M (1993). Rodent population dynamics in a primeval deciduous forest (Białowieża National Park) in relation to weather, seed crop and predation. Acta Theriol.

[54] Overgaauw PAM, van Knapen F (2008). Toxocarosis, an important zoonosis. EJCAP.

[55] Otranto D, Deplazes P (2019). Zoonotic nematodes of wild carnivores. Int J Parasitol Parasites Wildl.

[56] Letkova V, Lazar P, Curlik J, Goldova M, Kocisova A, Kosuthova L (2006). The red fox (*Vulpes vulpes* L.) as a source of zoonoses. Vet Athiv..

[57] Ziadinov I, Deplazes P, Mathis A, Mutunova B, Abdykerimov K, Nurgaziev R (2010). Frequency distribution of *Echinococcus multilocularis* and other helminths of foxes in Kyrgyzstan. Vet Parasitol.

[58] Gicik Y, Kara M, Sari BI, Kilic K, Arslan O (2009). Intestinal parasites of red foxes (*Vulpes vulpes*) and their zoonotic importance for humans in Kars province. KAÜ Veteriner Fakültesi.

[59] Borecka A, Gawor J, Zięba F (2013). A survey of intestinal helminths in wild carnivores from the Tatra National Park, southern Poland. Ann Parasitol.

[60] Borecka A, Gawor J (2009). Helminth intestine fauna in foxes from central Poland. Mag Wet.

[61] Vervaeke M, Dorny P, de Bruyn L, Vercammen F, Jordaens K, van den Berge K (2005). A survey of intestinal helminths of red foxes (*Vulpes vulpes*) in northern Belgium. Acta Parasitol.

[62] Goszczyński J. Fox. Natural and hunting monograph. Warsaw: Oikos; 1995

[63] Goszczyński J (1977). Connection between predatory birds and mammals and their prey. Acta Theriol.

[64] Robertson RA, Whelan J (1987). The food of the red fox (*Vulpes vulpes*) in Co. Kildare, Ireland. J Zool.

[65] Voigt DR (1987). Red fox.

[66] Dybing NA, Fleming PA, Adams PJ (2013). Environmental conditions predict helminth prevalence in red foxes in Western Australia. Int J Parasitol Parasites Wildl.

[67] Eslahi AV, Badri M, Khorshidi A, Majidiani H, Hooshmand E, Hosseini H (2020). Prevalence of *Toxocara* and *Toxascaris* infection among human and animals in Iran with meta-analysis approach. BMC Infect Dis.

[68] Mork T, Ims RA, Killengreen ST (2019). Rodent population cycle as a determinant of gastrointestinal nematode abundance in a low-arctic population of the red fox. Int J Parasitol Parasites Wildl.

